# Supplementary figures and images for: Follicle-innervating Aδ-low threshold mechanoreceptive neurons form receptive fields through homotypic competition
Source: Neural Dev. 2023 Apr 27;18:2. doi: 10.1186/s13064-023-00170-2 (PMC10134579; doi:10.1186/s13064-023-00170-2)

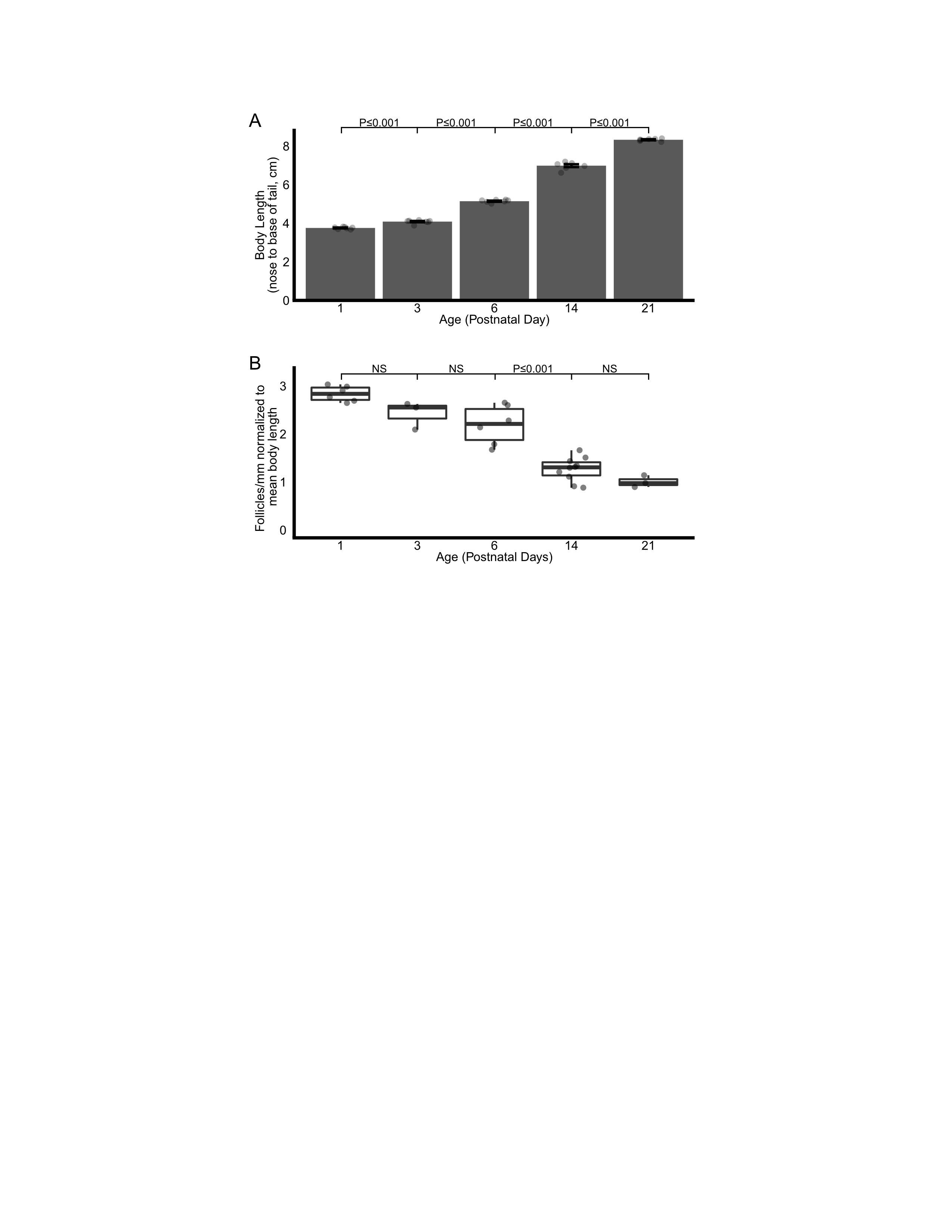

Supplement: Supplementary file 1 — Additional file 1: Supplemental Figure 1. Hair follicle density decreases relative to body length as mice grow. A) Average body length (nose to base of tail) of an age-matched cohort of 10 wild-type mice in centimeters. Bars represent mean ± s.e.m. One-way ANOVA with Tukey’s HSD post-hoc testing (ANOVA P≤ 0.001, P1-P3: P≤ 0.001, P3-P6: P≤ 0.001, P6-P14: P≤ 0.001, P14-P21: P≤0.001. B) Quantification of mouse hair follicle density from Figure 1H normalized to the mean body length of a cohort of 10 same-age mice shown in (A). Bars represent mean ± s.e.m. One-way ANOVA with Tukey’s HSD post-hoc testing (ANOVA P≤ 0.001, P6-P14 P≤ 0.001) N = 6(P1), 3(P3), 6(P6), 10(P14), 3(P21), 5(P60). [file 13064_2023_170_MOESM1_ESM.tiff]

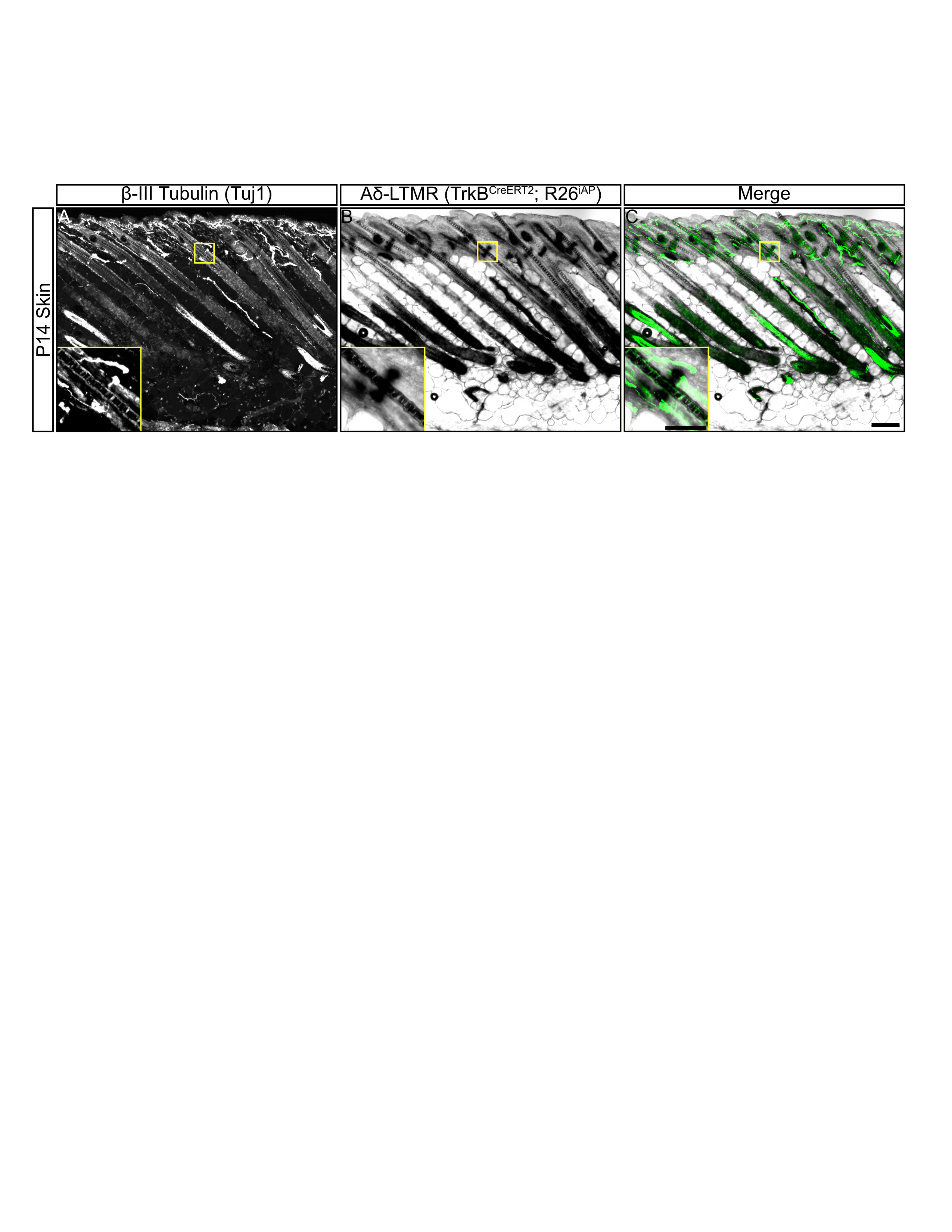

Supplement: Supplementary file 2 — Additional file 2: Supplemental Figure 2. Aδ-LTMR neurons fully innervate hair follicles by P14. A-C) Representative image of sagittal plane skin sections from a P14 mouse labeled with markers for all neurons (β-III Tubulin, A), Aδ-LTMRs (TrkBCreERT2;iAP), B), and merged channels (C). Insets – Higher magnification images of a β-III Tubulin+/TrkBCreERT2+ double positive longitudinal lanceolate ending. 92.2% ± 3% of follicles were innervated by a β-III Tubulin+/TrkB+ LLE. This is similar to previously reported values. N = 4 animals. Scale bar – 100 μm, 50 μm inset. [file 13064_2023_170_MOESM2_ESM.tiff]

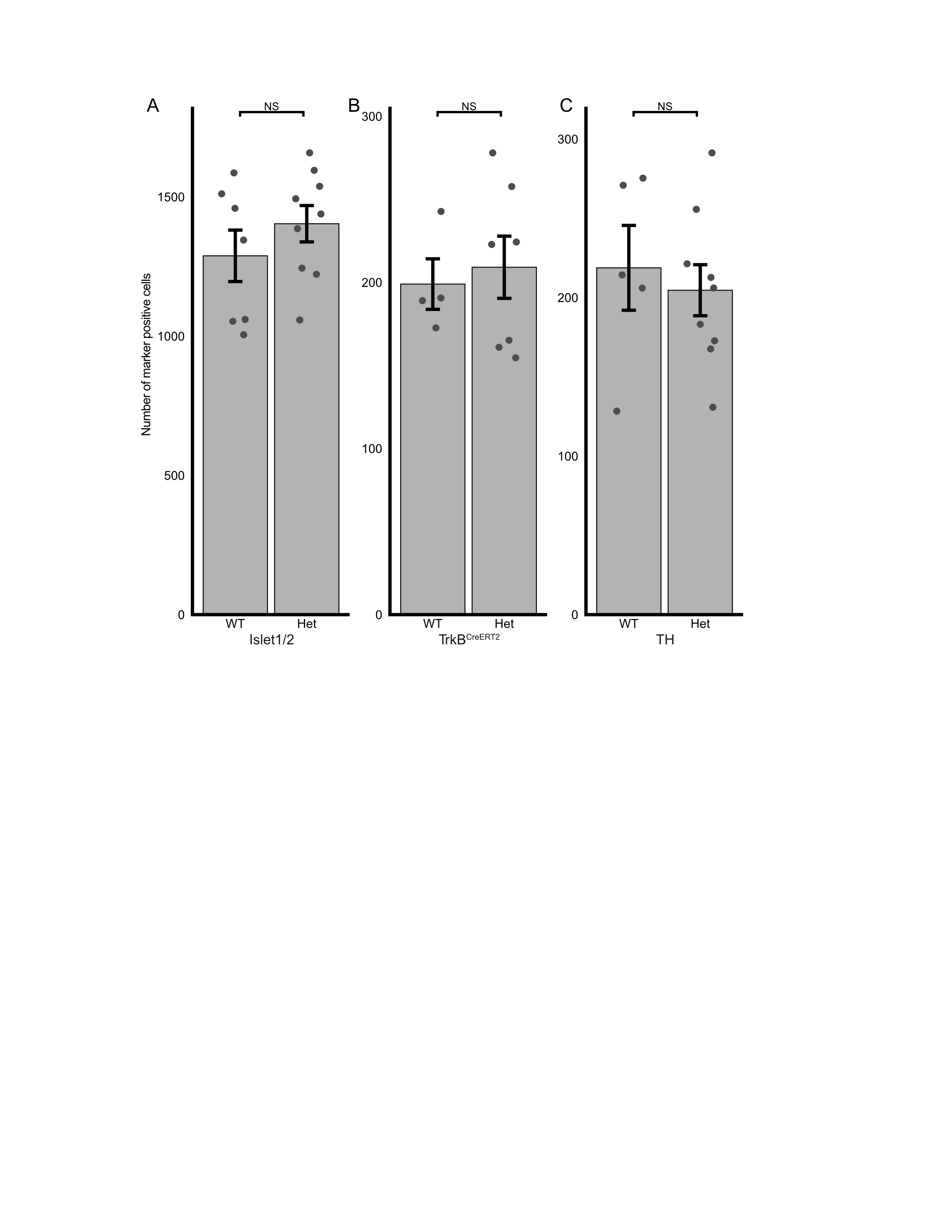

Supplement: Supplementary file 3 — Additional file 3: Supplemental Figure 3. Mice with heterozygous Bax deletion have no significant change in the number of neuronal nuclei, Aδ-LTMRs or C-LTMRs compared to wild-type mice. A) Comparison of Islet 1/2+ nuclei in Bax wild-type and heterozygous mice shows no significant difference. Bars represent mean ± s.e.m. N = 7(WT), 9(Het). Wilcoxon rank sum P≥ 0.05. B) Comparison of TrkBCreERT2+ cell bodies in Bax wild-type and heterozygous mice shows no significant difference. Bars represent mean ± s.e.m. N = 4(WT), 7(Het). Wilcoxon rank sum P≥ 0.05. C) Comparison of TH+ cell bodies in Bax wild-type and heterozygous mice shows no significant difference. Bars represent mean ± s.e.m. N = 5(WT), 9(Het). Wilcoxon rank sum P≥ 0.05. [file 13064_2023_170_MOESM3_ESM.tiff]

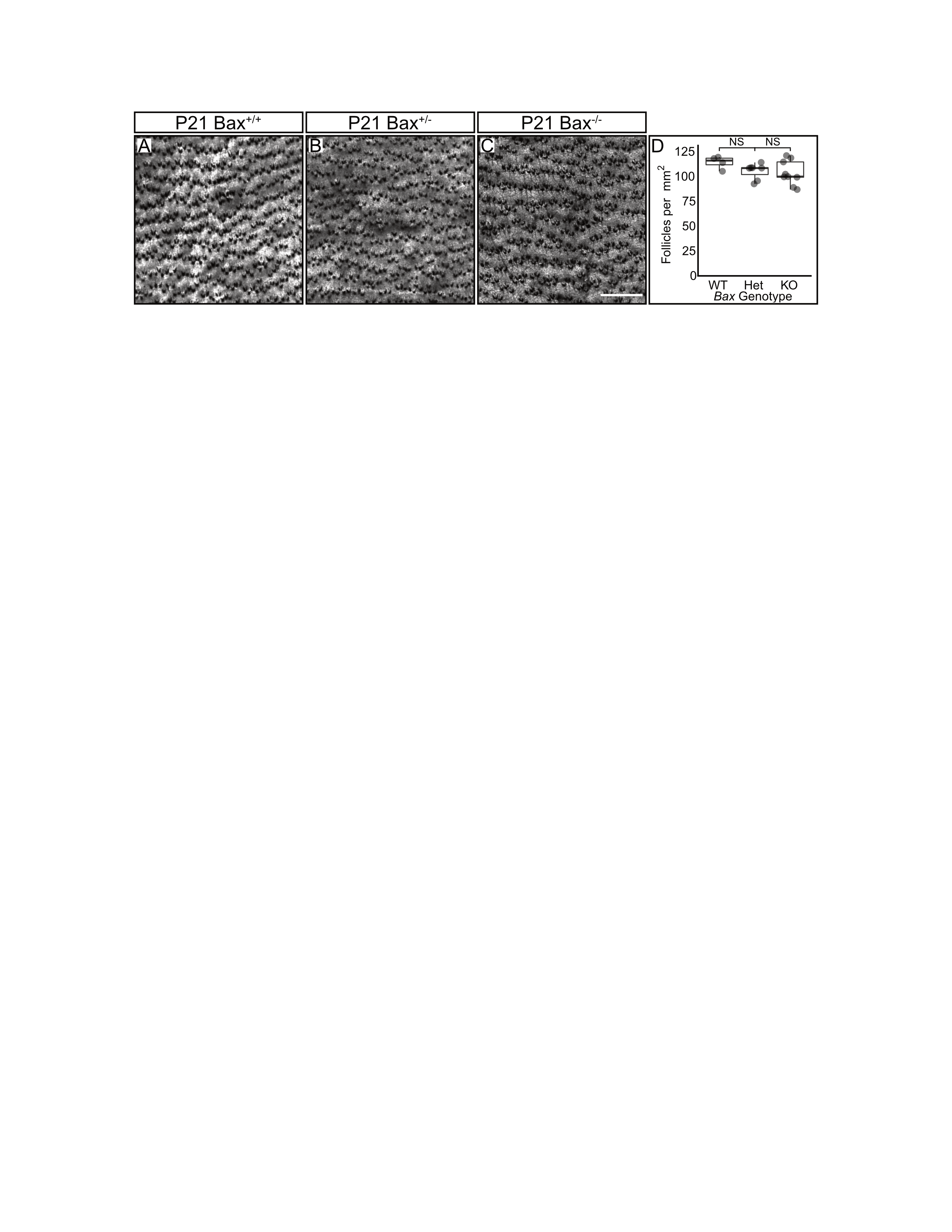

Supplement: Supplementary file 4 — Additional file 4: Supplemental Figure 4. Bax deficiency does not change hair follicle density. A-C) Representative images of P21 mouse back skin from Bax wild-type (A), heterozygous (B), and knockout (C) animals stained with the lipophilic marker Oil Red O to identify hair follicles. Scale bar – 500 μm. D) Quantification of the numbers of hair follicles per square millimeter of back skin. Wild-type: 113.2 ± 3.2 s.e.m. follicles/mm2, Heterozygous: 104.3 ± 3 s.e.m. follicles/mm2, Knockout: 102.5 ± 4.1 s.e.m. follicles/mm2. N = 4(WT), 7(Het), 9(KO). One-way ANOVA P= 0.22. [file 13064_2023_170_MOESM4_ESM.tiff]

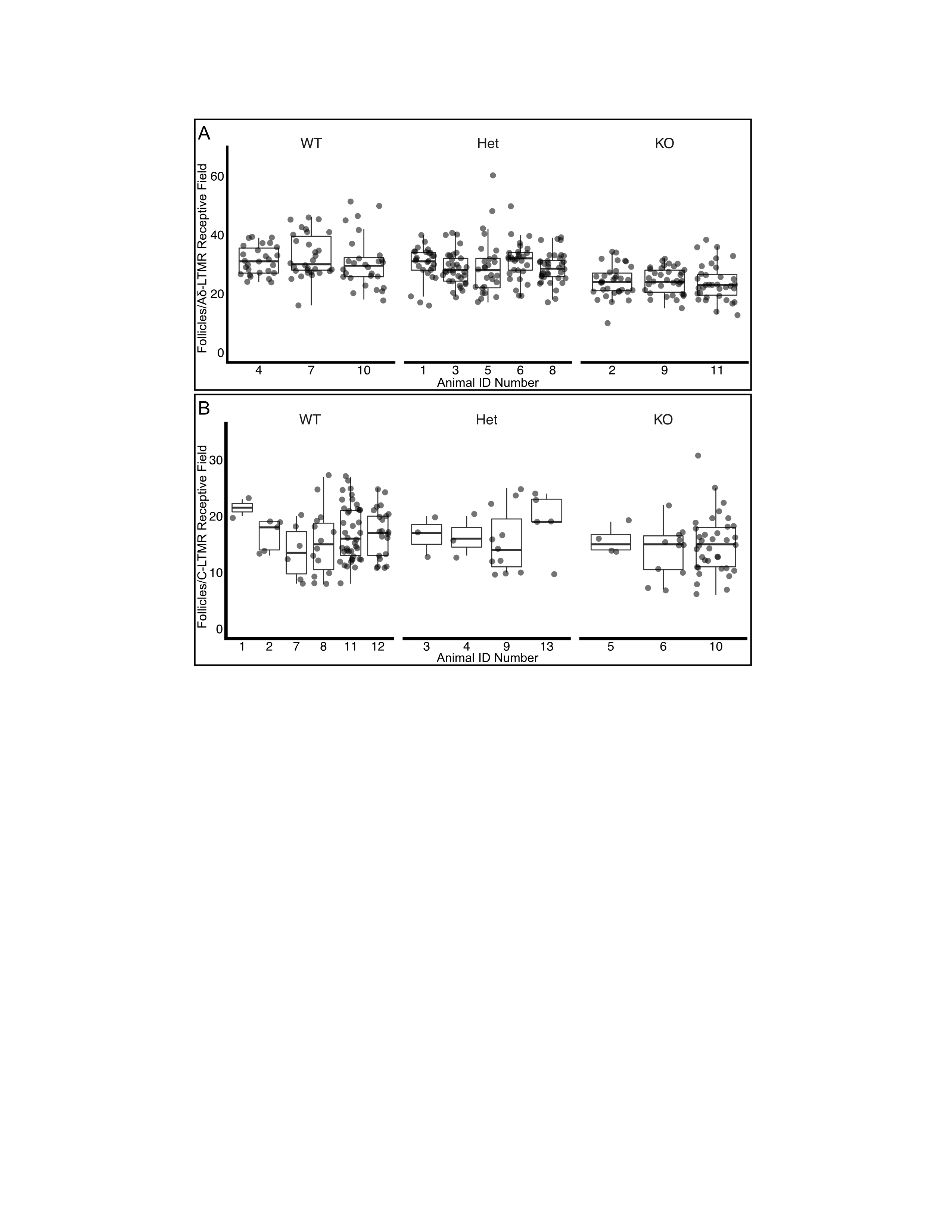

Supplement: Supplementary file 5 — Additional file 5: Supplemental Figure 5. Lack of inter-animal variation in follicles innervated per receptive field between same-genotype animals. A) Quantification of innervated hair follicles per Aδ-LTMR receptive field separated by animal. Each dot represents one quantified receptive field. No significant differences in number of innervated follicles per receptive field were seen between animals within genotype groups. Statistical analysis – One-way ANOVA Bax WT: P= 0.52, Bax Het: P= 0.49, Bax KO: P= 0.86. B) Quantification of innervated hair follicles per C-LTMR receptive field separated by animal. Each dot represents one quantified receptive field. No significant differences in number of innervated follicles per receptive field were seen between animals within genotype groups. Statistical analysis – One-way ANOVA Bax WT: P= 0.38, Bax Het: P= 0.70, Bax KO: P= 0.72. [file 13064_2023_170_MOESM5_ESM.tiff]
