# Supplementary material for: Follicle-innervating Aδ-low threshold mechanoreceptive neurons form receptive fields through homotypic competition
Source: Neural Dev. 2023 Apr 27;18:2. doi: 10.1186/s13064-023-00170-2 (PMC10134579; doi:10.1186/s13064-023-00170-2)
Supplement: Supplementary file 6 — Additional file 6: Supplementary Table 1. Summary follicle innervation data shown in Figures 1, 2 and 3. Supplementary Table 2. Summary Aδ-LTMR receptive field development data shown in Figure 4. Supplementary Table 3. Summary of Bax knockout DRG cell count data shown in Figure 5 and Supplementary Figure 3. [file 13064_2023_170_MOESM6_ESM.docx]

**Supplementary Table 1: Summary follicle innervation data shown in Figures 1-3.**

| Age | Follicles/mm  ± s.e.m.  (Fig. 1B-G) | % Tuj1^+^  ± s.e.m.  (Fig. 2A-F) | % Calbindin^+^  ± s.e.m.  (Fig. 3A-F) | % TH^+^  ± s.e.m.  (Fig. 3A-F) | % TrkB^+^/Tuj1^+^  ± s.e.m.  (Fig. 3A-F) |
| --- | --- | --- | --- | --- | --- |
| P1 | 10.66 ± 0.25 | 4.35 ± 1.28 | 0.05 ± 0.05 | 3.64 ± 0.59 |  |
| P3 | 9.90 ± 0.68 | 54.44 ± 1.50 | 20.16 ± 1.31 | 52.52 ± 2.03 |  |
| P6 | 11.25 ± 0.85 | 83.68 ± 6.17 | 15.44 ± 1.46 | 92.54 ± 1.26 |  |
| P14 | 8.85 ± 0.55 | 94.99 ± 0.94 | 17.07 ± 2.39 | 94.88 ± 1.07 | 92.2 ± 3.00 |
| P21 | 8.37 ± 0.60 | 94.33 ± 0.95 | 19.30 ± 2.95 | 94.15 ± 1.77 |  |
| P60 | 5.04 ± 0.48 | 90.69 ± 2.88 | 10.59 ± 1.83 | 97.61 ± 1.20 |  |

**Supplementary Table 2: Summary Aδ-LTMR receptive field development data shown in Figure 4.**

| Age | LLEs per Receptive Field  ± s.e.m. | Terminal Branches per Receptive Field ± s.e.m. |
| --- | --- | --- |
| P0/1 | 0.6 ± 0.08 | 26.2 ± 0.9 |
| P3 | 5.7 ± 0.5 | 26.0 ± 0.9 |
| P6 | 13.6 ± 0.8 | 27.8 ± 0.8 |
| P14 | 19.4 ± 0.8 | 20.8 ± 0.8 |

**Supplementary Table 3: Summary of Bax knockout DRG cell count data shown in Figure 5 and Supplementary Figure 3.**

| Cell Type | Marker | WT mean cell count ± s.e.m | Het mean cell count ± s.e.m. | KO mean cell count ± s.e.m. |
| --- | --- | --- | --- | --- |
| Neurons | Islet 1/2 | 1327.7 ± 98.9 | 1452.4 ± 74.3 | 1564.0 ± 98.0 |
| Aδ-LTMR | TrkB^CreERT2^ | 201.7 ± 21.2 | 229.6 ± 19.2 | 291.3 ± 40.7 |
| C-LTMR | TH | 205.8 ± 30.2 | 214.4 ± 19.3 | 279.5 ± 38.3 |
